# Evaluation of Therapeutic Efficacy and Imaging Capabilities of ^153^Sm_2_O_3_-Loaded Polystyrene Microspheres for Intra-Tumoural Radionuclide Therapy of Liver Cancer Using Sprague-Dawley Rat Model

**DOI:** 10.3390/pharmaceutics15020536

**Published:** 2023-02-06

**Authors:** Hun Yee Tan, Yin How Wong, Azahari Kasbollah, Mohammad Nazri Md Shah, Noorazrul Yahya, Basri Johan Jeet Abdullah, Chai Hong Yeong

**Affiliations:** 1 School of Biosciences, Faculty of Health and Medical Sciences, Taylor’s University, Subang Jaya 47500, Malaysia; 2 School of Medicine, Faculty of Health and Medical Sciences, Taylor’s University, Subang Jaya 47500, Malaysia; 3 Medical Advancement for Better Quality of Life Impact Lab, Taylor’s University, Subang Jaya 47500, Malaysia; 4 Medical Technology Division, Malaysian Nuclear Agency, Bangi 43000, Malaysia; 5 Department of Biomedical Imaging, University of Malaya Medical Centre, Kuala Lumpur 59100, Malaysia; 6 Diagnostic Imaging and Radiotherapy Programme, Faculty of Health Sciences, National University of Malaysia, Kuala Lumpur 50300, Malaysia

**Keywords:** samarium-153, polystyrene, microspheres, theranostics, liver tumour, Sprague-Dawley rat

## Abstract

**Introduction:** Neutron-activated samarium-153-oxide-loaded polystyrene ([^153^Sm]Sm_2_O_3_-PS) microspheres has been developed in previous study as a potential theranostic agent for hepatic radioembolization. In this study, the therapeutic efficacy and diagnostic imaging capabilities of the formulation was assessed using liver cancer Sprague-Dawley (SD) rat model. **Methods:** Twelve male SD rats (150–200 g) that implanted with N1-S1 hepatoma cell line orthotopically were divided into two groups (study versus control) to monitor the tumour growth along 60 days of treatment. The study group received an intra-tumoural injection of approximately 37 MBq of [^153^Sm]Sm_2_O_3_-PS microspheres, while control group received an intra-tumoural injection of 0.1 mL of saline solution. A clinical single photon emission computed tomography/computed tomography (SPECT/CT) system was used to scan the rats at Day 5 post-injection to investigate the diagnostic imaging capabilities of the microspheres. All rats were monitored for change in tumour volume using a portable ultrasound system throughout the study period. Histopathological examination (HPE) was performed after the rats were euthanized at Day 60. **Results:** At Day 60, no tumour was observed on the ultrasound images of all rats in the study group. In contrast, the tumour volumes in the control group were 24-fold larger compared to baseline. Statistically significant difference was observed in tumour volumes between the study and control groups (*p* < 0.05). The SPECT/CT images clearly displayed the location of [^153^Sm]Sm_2_O_3_-PS in the liver tumour of all rats at Day 5 post-injection. Additionally, the [^153^Sm]Sm_2_O_3_-PS microspheres was visible on the CT images and this has added to the benefits of ^153^Sm as a CT contrast agent. The HPE results showed that the [^153^Sm]Sm_2_O_3_-PS microspheres remained concentrated at the injection site with no tumour cells observed in the study group. **Conclusions:** Neutron-activated [^153^Sm]Sm_2_O_3_-PS microspheres demonstrated excellent therapeutic and diagnostic imaging capabilities for theranostic treatment of liver cancer in a SD rat model. Further studies with different animal and tumour models are planned to validate this finding.

## 1. Introduction

Primary liver cancer ranks the sixth most frequently diagnosed cancer and the third leading cause of cancer death worldwide, with an estimation of 906,000 new cases and 830,000 deaths in 2020 [1]. The high mortality rate of liver cancer is primarily due to late diagnosis. Intraarterial radioembolization, also known as selective internal radiation therapy (SIRT), is a recommended treatment for patients with unresectable liver tumours due to its established evidence in improving patient’s survival rate and quality-of-life [2]. In SIRT, millions of radioactive microspheres 20–60 µm in diameter are injected into the hepatic artery to deliver therapeutic beta (β^−^) radiations to liver tumours locally while sparing the adjacent healthy tissues [3,4]. 

The currently commercially available microspheres approved by the United States Food and Drug Administration (FDA) are SIR-Spheres^®^ (Sirtex Medical Ltd., Woburn, MA, USA), a resin-based microsphere and TheraSphere™ (Boston Scientific, Marlborough, MA, USA), a glass-beads microsphere [2]. Both SIR-Spheres^®^ and TheraSphere™ are labelled with radioactive Yttrium-90 (^90^Y), a pure beta (β^−^) emitter. One of the disadvantages of ^90^Y is the lack of gamma radiation which limits its application as a theranostic agent. Consequently, a surrogate radiopharmaceutical (such as ^99m^Tc-macroaggregated albumin) and imaging method (such as Bremsstrahlung imaging and positron emission tomography [PET]) are required for pre- and post-treatment imaging for ^90^Y-based radioembolization [5,6]. Therefore, some theranostic radiopharmaceuticals have been explored in the past to substitute ^90^Y-microspheres in liver radioembolization. For example, Holmium-166 (^166^Ho)-poly-L-lactic acid (PLLA) microsphere has received Conformité Européenne (CE) certification in 2015 under the commercial name QuiremSpheres™ (Quirem BV, Deventer, the Netherlands) [7]. There are some other potential theranostic radionuclides such as Iodine-131 (^131^I) and Rhenium-188 (^188^Re) [8,9,10]. However, both ^166^Ho and ^188^Re have short half-lives (26.8 h and 17.0 h, respectively), hence higher administered activities are needed which may cause unnecessary radiation damage to normal tissues and difficult for long distance transportation [6,11]. In contrary, ^131^I has a long physical half-life (8.02 days) and high gamma (γ) rays energy (E_γ_ = 364 keV) that raise a radiation safety concern to the patient as well as to the members of the public [6,11]. In addition, these high γ-rays energy are not ideal for imaging due to the poor count detection sensitivity thus resulting in image degradation [12]. There are studies using combination of two radionuclides with the theranostic concept where the γ-rays are used for diagnostics imaging or treatment planning and β^−^ particles for therapeutic such as Iodine-123 (^123^I)/^131^I and Galium-68 (^68^Ga)/Lutetium-177 (^177^Lu) for treatment of thyroid cancer and prostate cancer, respectively [13,14]. This idea of using more than one radionuclide has not been explored for liver cancer treatment.

In our previous study, a biocompatible polystyrene (PS) microspheres formulation loaded with non-radioactive samarium-152 oxide ([^152^Sm]Sm_2_O_3_) has been developed to overcome these limitations [15]. The microspheres are non-radioactive during synthesis until they are sent for neutron activation before the treatment. A large quantity of high specific activity samarium-153 (^153^Sm) can be produced via direct neutron capture process, ^152^Sm(n,γ)^153^Sm due to its high thermal neutron activation cross-section (206 barns) [16]. ^153^Sm has an optimal half-life of 46.3 h, low γ-ray energy (E_γ_ = 103 keV) for imaging and high β^−^ radiation (E_β_^−^_(max)_ = 808 keV) for therapy [17]. It has been used as a therapeutic agent for bone palliation and radiosynovectomy for more than 20 years [18]. Owing to its theranostics properties, ^153^Sm has a great potential in cancer management [19]. Personalized radiation therapy can be achieved with the presence of γ radiation that allows baseline and restaging imaging using the same radiopharmaceutical with to improve accuracy and safety of the treatment [20]. The purpose of the present study is to evaluate the therapeutic efficacy and diagnostic imaging capabilities of the [^153^Sm]Sm_2_O_3_-PS microspheres after intra-tumoural injection in liver tumour-bearing Sprague-Dawley (SD) rats.

## 2. Materials

Samarium oxide (Sm_2_O_3_) (99% purity), polyvinyl alcohol (PVA) (99% purity), chloroform, Cyclosporine A, meloxicam sodium salt hydrate (≥98% purity), formaldehyde (37%), sodium dihydrogen phosphate and disodium hydrogen phosphate were procured from Sigma Aldrich (St. Louis, MO, USA). Isoflurane USP was purchased from Piramal Healthcare Limited (Mumbai, India). The KTX (combination of 2.5 mL ketamine, 125 mg of tiletamine, 125 mg of zolazepam and 12.5 mL of xylazine) anesthesia was purchased from Laboratory Animal Resource Unit, National University of Malaysia (UKM), Kuala Lumpur, Malaysia.

## 3. Methods

### 3.1. Preparation of [^153^Sm]Sm_2_O_3_-PS Microspheres

The non-radioactive [^152^Sm]Sm_2_O_3_-PS microspheres with a mean diameter of 33.73 ± 0.23 µm were neutron-activated to [^153^Sm]Sm_2_O_3_-PS microspheres as previously described [15]. Several quality control tests were performed after neutron activation prior to the experiment. Firstly, the radioactivity of the neutron activated microspheres was measured and the specific activity obtained was similar to the previous study (5.04 ± 0.52 GBq·g^−1^). Radionuclide impurity was tested using gamma spectroscopy (Canberra, Meriden, CT, USA). In addition, the particle size and morphology of the microspheres were determined using a scanning electron microscope (SEM; Quanta 400, Hillsboro, OR, USA). Once the physicochemical properties were confirmed, the neutron-activated microspheres were suspended in saline solution to achieve a radioactivity of ~37 MBq per 0.1 mL of microspheres suspension.

### 3.2. Laboratory Animals

The animal experiments were approved by the National University of Malaysia Animal Ethical Committee (UKMAEC) (FSK/2019/Noorazrul/24-July/1017-Sept.-2019-Aug.-2021). A total of 18 healthy male SD rats (150–200 g) supplied by a local supplier (Chenur Supplier, Seri Kembangan, Selangor, Malaysia) were used in this study. The rats were bred on standard pelleted rat maintenance diet (PicoLab^®^ Rodent Diet 20 5053 Irradiated, LabDiet^®^, Richmond, IN, USA) and water ad libitum with 12 h light–dark cycle. 

### 3.3. Tumour Cell Line

The N1-S1 (ATCC^®^ CRL-1604™) hepatoma cell line was obtained from American Type Culture Collection (ATCC, Rockville, MD, USA) and was used for tumour implantation. The hepatoma cells were routinely cultured in Iscove’s Modified Dulbecco’s Medium (IMDM) (American Type Culture Collection, Rockville, MD, USA) supplemented with 10% fetal bovine serum (Gibco™, Thermo Fisher Scientific Inc., Grand Island, NY, USA) at 37 °C in 5% carbon dioxide (CO_2_) incubator (RS Biotech Galaxy S, Richmond Scientific Ltd., Chorley, England). A concentration of approximately 10^6^ to 10^7^ cells/mL was obtained after growing exponentially for 1 to 2 weeks when cell confluency reached approximately 90%.

### 3.4. Tumour Implantation

Cyclosporine A at a dose of 20 mg·kg^−1^ was administered subcutaneously 1 day before and 1 day after the tumour implantation to prevent spontaneous tumour regression of the N1-S1 hepatoma. Prior to the operation, the study and control groups rats were anesthetized using isoflurane through inhalation and meloxicam at a dose of 0.1 mg/100 g of body weight was administered subcutaneously for pain relief and reduce inflammation. Eye gel (GenTeal Gel, Alcon, Fort Worth, TX, USA) was applied to prevent eye dryness throughout the operation. Aseptic surgical techniques were used on all the rats. A subxiphoid laparotomy of about 1.5 to 2.0 cm in length was performed to expose the liver lobes. A hepatoma cell suspension containing 8 × 10^6^ cells in 20 µL was injected slowly using a 27 G needle into the lobus hepatis dexter medialis. Injection of the hepatoma cell suspension produced a visible pale-yellow region at the injection site. Post-injection, the needle was withdrawn slowly and pressed gently with a cotton to prevent bleeding and reflux of the cells. The incision wound was closed using a braided absorbable suture in layers and the rats were bandaged properly before placing them back in their cages.

### 3.5. Monitoring of Tumour Volume

After 8 days of hepatoma cell line injection, the tumour growth was monitored using a portable ultrasound with L12-4 broadband linear array transducer (Lumify, Philips Healthcare, Amsterdam, The Netherlands). Prior to ultrasound scanning, the rats were anesthetized by inhaled isoflurane. The tumour volume was monitored at pre-determined time points: before injection (Day 0), Day 14, Day 28, Day 42, and Day 60 after administration of microspheres or saline to monitor progression of liver tumours and to assess the therapeutic effects. The largest tumour diameter in mm, a and the smallest tumour diameter in mm, b were measured to determine the tumour volume (mm^3^) of the N1-S1 hepatoma-bearing rats that calculated by using the Equation (1) [21].

(1) $$\mathrm{Tumour}\;\mathrm{volume}=\frac{{a\;\times \;b}^{2}}{2}$$

The changes of tumour volume from Day 0 until Day 60 after the treatment were used to evaluate the treatment outcome or response. The outcome was based on two categories: (1) good response (tumour volume reduced by more than 10% or tumour disappeared) and (2) poor response (any condition less than a good response). 

### 3.6. Intra-Tumoural Injection of [^153^Sm]Sm_2_O_3_-PS Microspheres and Saline Solution

12 SD rats bearing with hepatic tumours were divided equally into two groups (study and control groups). The rats were given inhalational anesthesia before subxiphoid laparotomy (length between 1.5 and 2 cm). The animal preparation procedures are as described earlier. An intra-tumoural injection of ~37 MBq [^153^Sm]Sm_2_O_3_-PS microspheres was administered slowly into the center of the tumour of each rat in the study group [22,23,24], while 0.1 mL saline solution was injected to the center of the tumour in the control group. The puncture site was then gently pressed with cotton bud for 30 s to stop bleeding and the incision wound was closed in layers using braided absorbable suture. The rats were then bandaged properly and returned to their cages, respectively. Another six healthy rats without any surgical procedure were used as the healthy control.

### 3.7. SPECT/CT Imaging 

A clinical SPECT/CT system (BrightView XCT, Philips Healthcare, Hampshire, England) was used to scan the rats at Day 5 post-intra-tumoural injection. SPECT imaging was performed using a low-energy high-resolution collimator, image matrix of 128 × 128 and field of view (FOV) of 16 steps (20 s per step). CT images were acquired immediately after SPECT imaging using the following parameters: 120 kVp, 20 mAs, 3 to 4 min per scan, 256 × 256 image matrix, and 5 mm slice thickness. The rats were sedated by intraperitoneal injection of KTX at a dose of 0.1 mL per 100 g body weight to keep them in stable position throughout the imaging process. Additionally, whole body CT scans (Discovery MI DR, GE Healthcare, Chicago, IL, USA) were performed at Day 3 and Day 60 post-injection to detect the location of the microspheres in the rat’s liver. 

### 3.8. Histopathological Evaluation

After 60 days, all rats were euthanized by cervical dislocation after anesthetized with chloroform. The abdominal organs (liver, stomach, intestines, kidneys, and spleen) were examined for any abnormalities before dissecting out the liver. The dissected liver was fixed in 10% neutral buffered formalin for at least 48 h. The liver tissues were then trimmed along the long axis of the injection. The fixed tissues were then placed in a tissue cassette for tissue processing. The tissues were first embedded using paraffin-embedded technique and subsequently sliced into tissue slide with the thickness of 3 µm using a microtome. The tissue section was then stained with hematoxylin and eosin.

All histopathological examination (HPE) were conducted by an experienced pathologist with a light microscopy. The whole tissue sections were examined at low and high magnifications to describe the histopathological lesions with emphasis to the lesion severity. The evaluations of the tissue response on the microspheres were studied from the tissue samples obtained close to the implantation site at the liver lobe. The severity was described as minimal, mild, moderate, severe, and very severe.

### 3.9. Statistical Analysis

The data of the tumour volume were expressed as mean ± standard deviation. Mann-Whitney U test was used to compare the tumour volume between the study and control groups for each pre-determined time point. 95% confidence level was used and a *p*-value < 0.05 is considered as statistically significant difference.

## 4. Results

### 4.1. Therapeutic Efficacy of [^153^Sm]Sm_2_O_3_-PS Microspheres

**Figure 1:**
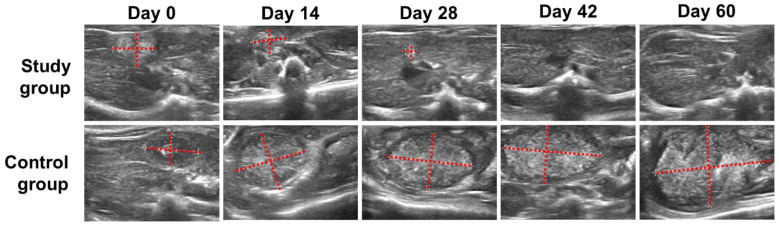
Ultrasound images of the rats in the study and control groups show tumour progression at Day 0, Day 14, Day 28, Day 42, and Day 60 post-injection of [^153^Sm]Sm_2_O_3_-PS microspheres and saline solution. The red dotted lines show the horizontal and vertical diameters of the tumour.

Figure 1 shows the ultrasound images of the rats in study and control groups at Day 0, Day 14, Day 28, Day 42, and Day 60 post-injection. The rats in study group, which had been administered with [^153^Sm]Sm_2_O_3_-PS microspheres, showed a more pronounced reduction from Day 0 to Day 14 followed by a gradual reduction from Day 14 to Day 42. At Day 28, 83% of rats showed a significant decrease in tumour size after intra-tumoral injection of [^153^Sm]Sm_2_O_3_-PS microspheres while the tumour was not observed in one of the rats. At Day 42, tumours were not observed in 33% of the rats and most of the rats showed a good tumour response. At Day 60, no tumour was observed on the ultrasound images of all rats in the study group. In contrast to the control group, the tumour volume was increased progressively over time.

**Figure 2:**
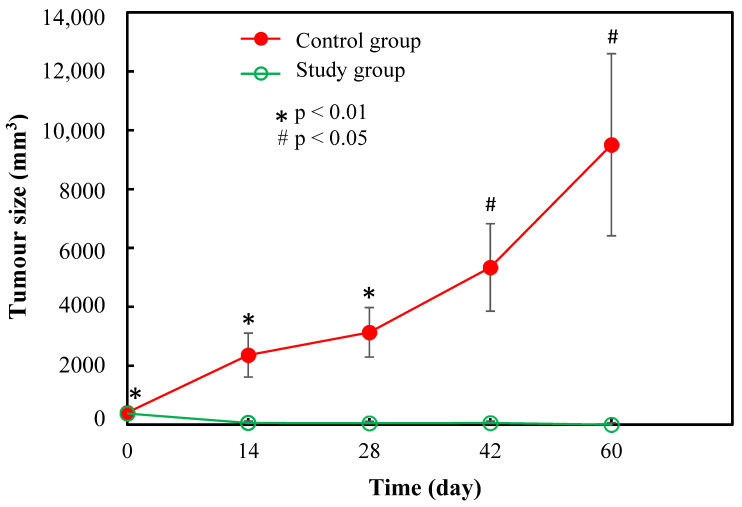
Tumour volume assessment of the effect of [^153^Sm]Sm_2_O_3_-PS microspheres on the N1-S1 hepatoma cell-induced tumour in SD rats. Tumour volume of the study and control groups were assessed at Day 0, Day 14, Day 28, Day 42, and Day 60 post-injection.

Figure 2 shows the changes of tumour volume over the period of 60 days in the study and control groups. The average tumour volume in the study and control groups at Day 0 (before intra-tumoural injection of microspheres or saline solution) were 372 ± 57 mm^3^ and 391 ± 88 mm^3^, respectively. The tumours in the control group showed a progressive growth from 391 ± 88 mm^3^ at Day 0 to 9507 ± 3095 mm^3^ at Day 60, which was about 24-fold larger compared to baseline. Statistically significant difference was observed in tumour volume between the study and control groups (*p* < 0.05). All rats in the study group (100%) showed good response after treated with the [^153^Sm]Sm_2_O_3_-PS microspheres while all rats in the control group (100%) showed poor response. 

### 4.2. Diagnostic Imaging Capabilities of [^153^Sm]Sm_2_O_3_-PS Microspheres

**Figure 3:**
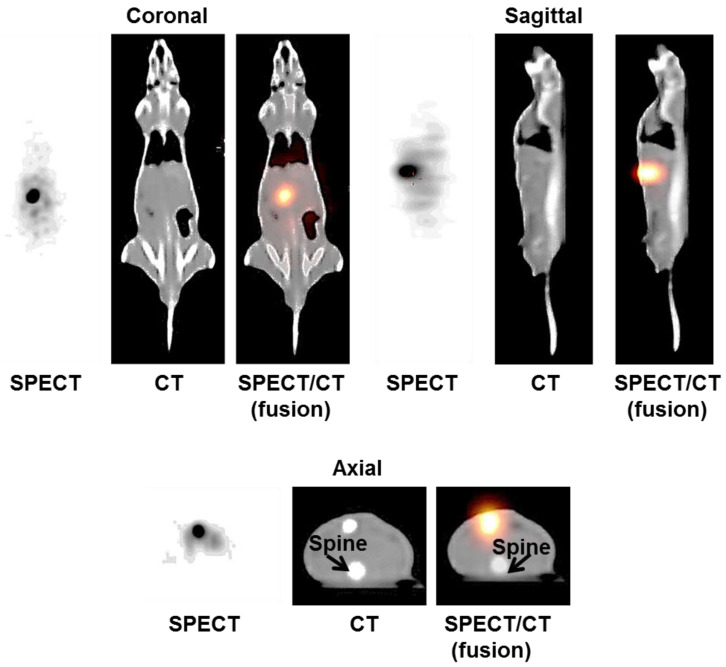
SPECT/CT images of a rat in the study group at Day 5 post-injection. Images displayed the location of ^153^Sm microspheres in the liver tumour.

The SPECT, CT, and hybrid SPECT/CT images of a rat in the study group are presented in Figure 3. As shown in the figure, the radioactive [^153^Sm]Sm_2_O_3_-PS microspheres accumulated at the injection site with no detectable leakage of the microspheres in other organs. 

**Figure 4:**
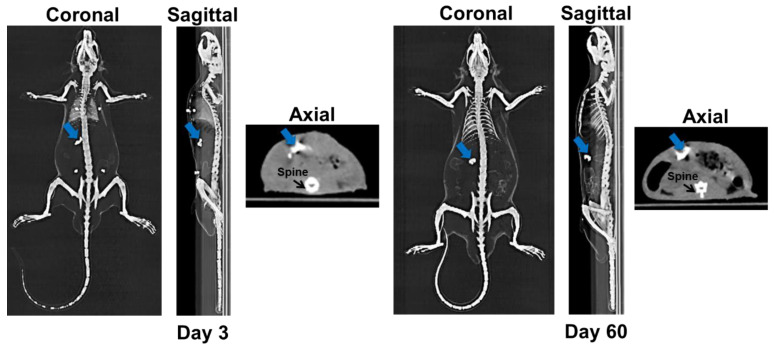
CT images of the rats in the study group at Day 3 and Day 60 post-injection of the [^153^Sm]Sm_2_O_3_-PS microspheres. The blue arrow indicates the location of the microspheres.

In addition to the gamma imaging capability, the [^153^Sm]Sm_2_O_3_-PS microspheres were also visible on the CT images as a radiopaque agent. The location of the microspheres within the tumour can be seen clearly on the CT images acquired at Day 3 and Day 60 post-injection as shown in Figure 4. The CT number of the microspheres was 817.24 ± 49.55 HU which was higher than the CT number of the normal liver (40.41 ± 1.83 HU). The CT number of the microspheres was found comparable to the CT number of the bone at 709.37 ± 36.37 HU.

### 4.3. Histopathological Examinations

**Figure 5:**
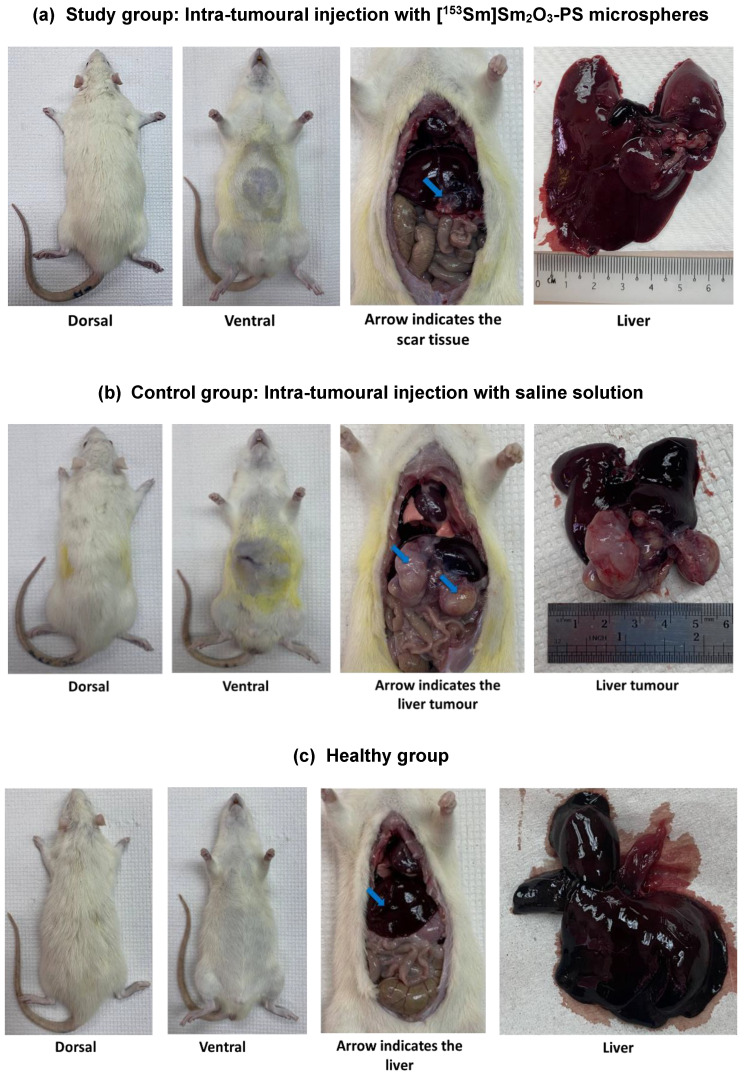
The dorsal view, ventral view, and abdominal organs for each rat in (**a**) study, (**b**) control, and (**c**) healthy groups that were sacrificed at Day 60.

After 60 days, all the rats in the study, control, and healthy groups were sacrificed for HPE assessment. The rats from the study (Figure 5a) and healthy (Figure 5c) groups appeared to be healthy and normal at the end of 60-day monitoring period while the rats in the control group showed enlarged abdomen due to the liver tumours (Figure 5b). On visual inspection of the abdomen and thoracic organs of the healthy group upon dissection, no abnormality was observed. The abdomen and thoracic organs of the rats in the study group appeared to be normal. The macroscopic inspection of the liver of the rats in the study group showed the liver’s laceration replaced with scar tissues (Figure 5a). In contrast, large liver tumours were observed on the rats in the control group and in some of the rats, liver metastases were also observed (Figure 5b).

**Figure 6:**
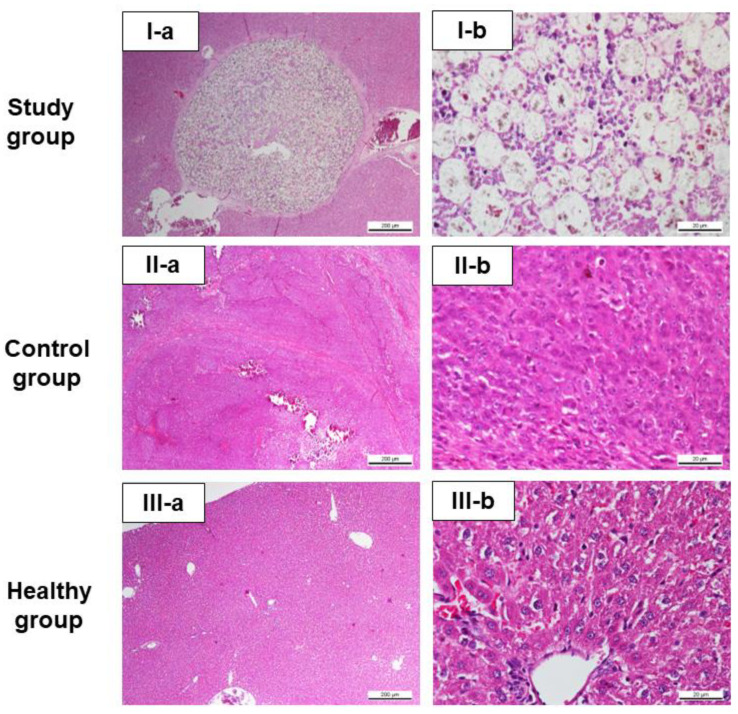
Microphotograph of liver tissue section of the representative rats in all three groups. (**I-a**,**I-b**) Study group. (**II-a**,**II-b**) Control group. (**III-a**,**III-b**) Healthy group.

Figure 6 shows the cross-sectional microscope images of the liver tissue in all three groups. For the study group, the liver section generally shows normal liver, except for the presence of focal hepatitis, and a large area containing numerous microspheres. The microspheres were completely encapsulated by moderate thickness of fibrous tissue with scattering lymphocytes. Numerous viable and non-viable inflammatory cells detected at the vicinity of the microspheres and there are no residual tumour cells observed in the study group at Day 60 post-treatment, as shown in Figure 6I-a,I-b. In contrast, large liver tumour was observed during post-mortem of the control group, and some rats had developed extensive liver metastases. More than 90% of the sampled liver tissues in the control group showed lesions which mainly consisted of neoplastic cells arranged in large coalescing islands. These islands showed evidence of central necrosis with extensive calcification, with moderate hemorrhages (Figure 6II-a). At higher magnification, the mitotic figures were 9.90 ± 6.37 per high power field. These neoplastic cells showed large nuclei with prominent nucleoli of various sizes and shape. Binucleation was occasionally seen, and the tumour is consistent with hepatocellular carcinoma cells showed mitotic figures Figure 6II-b). As for the healthy group, observation of normal hepatic section with normal hepatocytes and arranged in normal architecture were shown in Figure 6III-a,III-b.

## 5. Discussion

The radioactive [^153^Sm]Sm_2_O_3_-PS microspheres, a relatively novel formulation of theranostic radiopharmaceutical had been developed as a potential radioembolization agent for liver cancer. In order to investigate the therapeutic and diagnostic imaging capabilities of the microspheres, the microspheres were administered intra-tumourally into the liver tumour of the SD rat model. The radiation damage to the surrounding healthy liver tissues is negligible as ^153^Sm emits medium energy β^−^ particles, which have a mean tissue penetration of 0.8 mm and a maximum penetration of 4.0 mm. The low energy γ-rays (103 keV) only contributes to about 28% of the overall decay but it is useful for diagnostic imaging using a gamma camera. The γ imaging capabilities of ^153^Sm has been shown in the previous studies to investigate gastrointestinal motility using ^153^Sm-loaded radiotracer [25]. This is evident by little calcifications and necrotic tissues debris found at the adjacent liver tissues using a light microscope, however no hepatotoxicity was reported in all the treated rats. 

Owing to the favourable imaging properties of ^153^Sm, SPECT imaging can be performed to assess the biodistribution of the [^153^Sm]Sm_2_O_3_-PS microspheres. The microspheres were retained at the tumour site that can be clearly visualized on the SPECT/CT images at Day 5 post-injection. Additionally, the microspheres were visible significantly on the CT images taken at Day 3 and Day 60 post-injection. This is a remarkable finding and added to the benefit of ^153^Sm as a CT contrast agent. A study by Nakayama et al. [26] confirmed that the CT numbers of samarium doped titanium dioxide nanoparticles were significantly higher than those without samarium due to its high atomic number (Z).

After 60 days, it was observed from the HPE that a large number of microspheres were still located at the injected site with no tumour cells detected. The thick fibrous tissue and a small area of scar tissue at the injected site were observed visually in some of the study group rats. This may be due to radiation necrosis or injury during intra-tumoural administration of the microspheres. All the rats in the study group achieved good response and the complete responses were confirmed by HPE which showed no viable tumour tissue and abnormalities (inflammation or lesions) in the liver samples at Day 60. However, all the rats in the control group which were injected with saline solution showed poor responses as the tumours progressively increased in size. 

In this experiment, a single fixed-volume injection of [^153^Sm]Sm_2_O_3_-PS microspheres was used, and the microspheres were injected into the central area of the tumour. Because ^153^Sm has a mean tissue penetration of 0.8 mm and a maximum penetration of 4.0 mm, the positive therapeutic effects are highly dependent on the shape of the tumour. In the case when the tumour has an irregular shape, multiple injections may be more effective than a single injection as suggested by Junfeng et al. [27]. An accurately calculated dose of [^153^Sm]Sm_2_O_3_-PS microspheres depending on the size and shape of the tumour, as well as the normal liver function should be able to increase the treatment efficacy, however this is not within the scope of this study. Future studies should include radiation dosimetry to improve the treatment outcomes.

In a study conducted by Wang et al. [22] that used ^188^Re-labelled microspheres to treat hepatoma in a rat model, 10 out of 15 rats showed good response to the treatment, and the response was noticeable at Day 14 continued until Day 28 post-injection, which was similar to the finding of this study. However, only 67% of the rats in Wang et al. study [22] showed significant reduction in tumour size, of which 20% showed complete disappearance of tumour at 60 days post-treatment. 5 out of 15 rats showed poor response, including 3 rats died during the study and 2 other rats showed good response in the second week but with tumour rebound in the fourth week. In another study using ^188^Re microspheres, only 52% of the rats showed good response in the study group [21]. However, complete tumour disappearance only observed at Day 60 post-injection of the microspheres. All the studies mentioned above showed poor responses in the control group injected with saline solution that expressed the similar results of this study.

The treatment with ^188^Re microspheres in the previous studies showed only 50–60% good response rate, compared to ^153^Sm microspheres in this study which achieved 100% good response rate. This might be due to the shorter half-life of ^188^Re compared to ^153^Sm (17.0 h versus 46.3 h). The injected radioactivity of ~37 MBq ^188^Re microspheres were fully decayed in a relatively shorter duration, hence reducing its efficacy in tumour cells necrosis. There are many factors affect the therapeutic efficacy and particularly the rate of β^−^ particles emission which is associated to half-life and the number of β^−^ particles which is associated to the radioactivity [28]. The biological effect of radiations is determined by the absorbed dose (Gy) that described by the energy (J) deposited per mass of tissue or tumour (kg) where this absorbed dose is directly related to the tumour response and cell survival fraction [29]. A radionuclide half-life should correspond to pharmacokinetics of the carrier in-vivo. This indicates that the half-life must be optimum and longer than the time needed to prepare the radiopharmaceutical, for delivery to site, administration, and biodistribution in a tumour [30]. 

As reported by Lin et al. [23], 10 out of 12 rats treated by intra-tumoural injection of ^90^Y microspheres revealed good response to the treatment with tumour completely disappeared in 5 rats at Day 14 but 2 rats showed poor response to the treatment. The response to saline solution was poor in all 12 rats in the control group. In comparison, treatment by ^131^I microspheres resulted in smaller tumour volume at each time point compared to the control group, beginning at Day 7 up to Day 60 post-injection. In the study group treated with ^131^I microspheres, the tumour volume was 200% smaller than that before injection and complete tumours disappearance was observed in 2 rats at Day 14 [24]. Both studies showed faster response rate in the study group (Day 14) compared to this study where complete tumour disappearance in 1 rat was only noticed at Day 28 post-injection. This might be due to the higher β^−^ energy emitted from ^90^Y microspheres (2.28 MeV). Both studies showed poor response in the control group that expressed the same outcomes from this study. 

Intra-tumoural injection of radiopharmaceutical for liver cancer treatment has been conducted in the past 20 years as a promising approach in treating unresectable solid liver tumours [31,32,33,34,35]. As reported by Dong et al. [31], all 28 hepatic carcinoma patients treated with percutaneous injection of [^90^Y]-glass microspheres survived during 2–16 months of follow-up. The tumour volume reduction achieved 91% and all patients experienced relief of symptoms as well as improvement in general conditions. Complete necrosis and fibrosis of the tumours were observed in HPE in 7 out of 8 patients after treatment, with only 1 patient showed a small focus of tumour tissue. Tian et al. [32] also performed intra-tumoural injection of [^90^Y]-glass microspheres on liver cancer patients and encouraging outcomes have been observed. The patients had dramatic improvement in their clinical conditions where 90.6% of the tumour foci became smaller, with echogenic or blood flow changes on liver sonograms. At the end of the study, 27 of 33 patients were still alive after treatment (12–32 months).

In Lee et al. [33] study, it was demonstrated [^166^Ho]Ho-macroaggregate or chitosan radioactivity was well accumulated within 56 hepatoma of 50 patients with no detectable extrahepatic uptake of radioactivity in lung, bowel, kidney and other organs. Overall, therapeutic effect was achieved in 41 patients (82%) where the tumours in 32 patients (64%) were completely treated, 7 patients had recurrence of treated tumour and failed in 2 patients. The same observation was found by Kim et al. [34] using [^166^Ho]Ho-chitosan with complete tumour necrosis was achieved in 11of 12 patients (91.7%) with HCC lesions < 2 cm and in 31 of 40 patients (77.5%) with HCC lesions < 3 cm after 2 months. Furthermore, the survival rates were 87.2%, 71.8%, and 65.3% at 1, 2, and 3 years, respectively. Throughout the long-term follow up period, tumours were recurred in 28 patients, of which 24 recurred at another intrahepatic site. Another clinical study using phosphorus-32 (^32^P) BioSilicon given intra-tumourally to patients with unresectable HCC showed all target tumours reduced in size at 12 weeks, with a complete response (100% regression) in 2 of the 8 patients [35]. In addition, further reductions were observed in 4 patients at 24 weeks and throughout the study period no clinically significant adverse events were noted. There was no detectable radioactivity in the patient’s blood directly after implantation of [^32^P]P-BioSilicon. 

The encouraging outcomes from this study, along with all the previous studies mentioned above indicated that intra-tumoural injection of radioactive microspheres is a potentially effective treatment in shrinking or killing liver cancer cells. High therapeutic radiation(s) can be delivered directly and localize at the tumour site with minimal or no leakage of radiation to the adjacent healthy tissues. This approach greatly improves treatment efficacy and safety (high tumour-to-normal irradiation ratio, less radiotoxicity to the healthy tissues, less complications or side effects, better quality-of-life). In addition, intra-tumoural injection is less personnel dependence compared to intra-arterial delivery that requires interventional radiologist’s skill and special equipment for precise catheterization [32]. Moreover, some patients may have high lung shunting fraction and therefore are not suitable for intra-arterial administration. Hence, one possible option to administer the radiopharmaceuticals for these patients is through intra-tumoural injection. The therapeutic efficacy outcome indicated that the intra-tumoural injection is an appropriate first-line treatment for patients with HCC as it was found to be a safe procedure and could be used as a bridge to liver transplantation. Further exploration needs to be carried out on the radiation safety of the intra-tumoural injection technique as well, especially to the personnel that handling the radioactive microspheres. Thus, more investigations on the technique are necessary before this procedure can be widely accepted and applied as an option for the treatment of liver cancer.

Despite the promising outcomes, the present study is limited by its small sample size (6 rats for each group) due to the recommendation from the animal ethics committee. Furthermore, the imaging modalities used in this study, including the portable ultrasound, SPECT/CT and CT scanners are clinical systems intended to use for human imaging, and hence the image resolution might be limited for small animals. For future animal studies, it is recommended to apply a larger sample size and to use pre-clinical imaging modalities designated for small animals imaging.

## 6. Conclusions

This study demonstrated that neutron-activated [^153^Sm]Sm_2_O_3_-PS microspheres is an effective therapeutic agent with potential imaging capabilities for liver cancer treatment tested in a SD rat model. A good response rate of 100% was achieved in the study group (n = 6) where all the liver tumours disappeared in all rats at 60 days post-treatment, with no hepatoxicity or other abnormalities seen in these rats. The [^153^Sm]Sm_2_O_3_-PS microspheres showed its imaging capabilities where the microspheres can be visualized on SPECT/CT and CT images. 

## Data Availability

No new data were created or analyzed in this study. Data sharing is not applicable to this article.
